# Hybrid-Pattern Recognition Modeling with Arrhythmia Signal Processing for Ubiquitous Health Management

**DOI:** 10.3390/s22020689

**Published:** 2022-01-17

**Authors:** Wei-Ting Hsiao, Yao-Chiang Kan, Chin-Chi Kuo, Yu-Chieh Kuo, Sin-Kuo Chai, Hsueh-Chun Lin

**Affiliations:** 1 Department and Institute of Health Service Administrations, China Medical University, Taichung 406040, Taiwan; u106026320@cmu.edu.tw (W.-T.H.); fish840319@gmail.com (Y.-C.K.); skchai@mail.cmu.edu.tw (S.-K.C.); 2 Department of Electrical Engineering, Yuan Ze University, Taoyuan 32003, Taiwan; yckan@saturn.yzu.edu.tw; 3 Division of Nephrology, Department of Internal Medicine, China Medical University Hospital, College of Medicine, China Medical University, Taichung 40402, Taiwan; fenderkuo@gmail.com; 4 Big Data Center, China Medical University Hospital, Taichung 40402, Taiwan

**Keywords:** Hilbert–Huang transform, empirical mode decomposition, intrinsic mode function, marginal Hilbert spectrum, multiclass recognition, machine learning, ubiquitous health management

## Abstract

We established a web-based ubiquitous health management (UHM) system, “ECG4UHM”, for processing ECG signals with AI-enabled models to recognize hybrid arrhythmia patterns, including atrial premature atrial complex (APC), atrial fibrillation (AFib), ventricular premature complex (VPC), and ventricular tachycardia (VT), versus normal sinus rhythm (NSR). The analytical model coupled machine learning methods, such as multiple layer perceptron (MLP), random forest (RF), support vector machine (SVM), and naive Bayes (NB), to process the hybrid patterns of four arrhythmia symptoms for AI computation. The data pre-processing used Hilbert–Huang transform (HHT) with empirical mode decomposition to calculate ECGs’ intrinsic mode functions (IMFs). The area centroids of the IMFs’ marginal Hilbert spectrum were suggested as the HHT-based features. We engaged the MATLAB^TM^ compiler and runtime server in the ECG4UHM to build the recognition modules for driving AI computation to identify the arrhythmia symptoms. The modeling extracted the crucial data sets from the MIT-BIH arrhythmia open database. The validated models, including the premature pattern (i.e., APC–VPC) and the fibril-rapid pattern (i.e., AFib–VT) against NSR, could reach the best area under the curve (AUC) of the receiver operating characteristic (ROC) of approximately 0.99. The models for all hybrid patterns, without VPC versus AFib and VT, achieved an average accuracy of approximately 90%. With the prediction test, the respective AUCs of the NSR and APC versus the AFib, VPC, and VT were 0.94 and 0.93 for the RF and SVM on average. The average accuracy and the AUC of the MLP, RF, and SVM models for APC–VT reached the value of 0.98. The self-developed system with AI computation modeling can be the backend of the intelligent social-health system that can recognize hybrid arrhythmia patterns in the UHM and home-isolated cares.

## 1. Introduction

In the artificial intelligence (AI) era, ubiquitous health management (UHM) and care services have been made to be compliant with smart medical techniques. With communication computing technology, physiological signals can be routinely tracked by wearable sensors at home for clinical diagnosis of chronic diseases (e.g., arrhythmia) and even home-isolated cares (e.g., COVID-19) [1,2]. Medical informatics with AI computation can be targeted in a recognition model to efficiently identify clinical data characteristics for health risk assessment and prevention [3,4,5]. The AI-enabled machine learning (ML) model typically consists of featuring and data training, which pre-processes the labeled samples (e.g., specific symptoms in the electrocardiogram (ECG) of arrhythmia) and explores the features to recognize clinical data [6,7,8]. Hybrid ML methods were qualified for coupling analysis of comprehensive features.

The neural network (NN) and dimension separation were both classification types of the typical ML methods. The multilayer perceptron (MLP) [9] was a well-known NN-based ML method that simulates the neurons in the biological nervous systems to enable the forward and backward iterations in the input, hidden, and output layers for training data. The dimension separation derives the mathematical formulations for recognizing the multiclass labels of the features in which the support vector machine (SVM) [10], naive Bayes (NB) classifier [7], and random forest (RF) for ensemble analysis [11] are popular for identifying biomedical data. With training of the clinical samples, both types of ML methods generated prediction models that recognize physiological signals and provide indicators of chronic diseases (e.g., arrhythmia ECG) [12]. A hospital can apply trained ML models in UHM using the Internet of Things (IoT) for intelligent social health services, such as a patient wearing a mobile ECG device at home and delivering daily measured signals to a cloud server through customized apps for AI computation and recognition of disease symptoms. The ML models in the present ubiquitous health (uHealth) scope that combines mobile functions with cloud services for the UHM are limited to a few arrhythmia samples for monitoring cardiovascular diseases [13,14,15].

A conventional 12-lead ECG examination measures the voltage of ten electrodes for six limb leads and six chest leads. The modified limb lead ECG is subjected to atrial activity enhancement during rest or exercise. The analytical signals are typically measured by the modified limb lead II (MLII), the voltage between the electrodes on the specific intercostal space to the left leg and the right arm [16]. Early studies proposed a three-dimensional dynamic model that mathematically formulated the heart rhythm to the space position and the time when a particle moves along the heart pulse waveforms [17]. At present, the MIT-BIH arrhythmia database provided by the Massachusetts Institute of Technology is a valuable open data source of ECGs for academic research [18,19]. The original source involved a set of over 4000 recordings by the Beth Israel Hospital Arrhythmia Laboratory from between 1975 and 1979. The analytical database contained 23 records (#100–#124) randomly chosen from this set, and 25 records (#200–#234) with various important phenomena. Every ECG data set with a sampling frequency of 360 Hz was recorded for approximately 30 min in the database. A variety of arrhythmia symptoms were annotated, such as atrial premature atrial complex (APC or PAC), atrial fibrillation (AFib), ventricular premature complex (VPC or PVC), and ventricular tachycardia (VT), in addition to normal sinus rhythm (NSR), which had the most records. Recent studies have utilized machine and deep learning to detect arrhythmia and displayed good recognition of a combination of specific symptoms [20,21].

The ECG signals usually involve time–domain features such as waveforms of PQRST waves and the intervals of PR, QRS, QT, ST, RR, and QRS complex waves. Following the international measurement standard of heart rate variability (HRV), the low and high frequencies of heart pulses were defined for analysis of power spectral density (PSD) to classify the features of heart rhythm in a frequency domain [22]. These past studies explored the HRV-related characteristics in both domains. In general, the time–domain approaches compared the PQRST wave positions to observe rhythm changes for clinical monitoring. The fast Fourier transform (FFT) is a well-known method to filter noise and calculate a spectrum for classifying frequency–domain features [23]. A wearable ECG device could help to identify the hybrid symptoms of arrhythmia while measuring HRV at home [24,25,26] and would improve UHM.

Moreover, the Hilbert–Huang transform (HHT) performs a process of empirical mode decomposition (EMD) to factorize nonlinear and non-steady signals into a combination of the intrinsic mode functions (IMFs) [27]. The order of computational complexity of the EMD is equivalent to FFT [28]. The IMF can further constitute the Hilbert spectrum (HS) including the instantaneous frequency and energy in the time–frequency domain. The HHT has been widely used for wavelet analysis of the electroencephalogram and ECG in biomedical engineering and informatics [29,30,31,32].

In this study, we developed a prototype of a UHM system (UHMS) and conducted an AI computation with multiclass recognition models composed of the ML methods (e.g., MLP, RF, SVM, and NB) and the HHT-based features to identify the hybrid patterns of four arrhythmia symptoms (APC, AFib, VPC, and VT) versus normal sinus rhythm (NSR). The system was constructed using the Java and MATLAB^TM^ languages.

## 2. Methods

**Figure 1:**
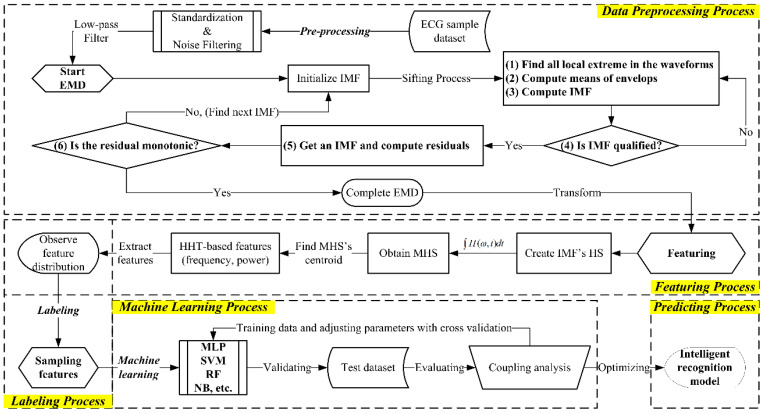
The AI-compliant modeling process with HHT-based featuring and coupling ML analysis for multiclass recognition of arrhythmia ECG data.

The MIT-BIH arrhythmia open database was used in this study to assess multiclass recognition modeling in the proposed UHMS. As shown in Figure 1, the AI-compliant computation processes four analytical slices including data pre-processing, featuring, labeling, and machine learning behind the UHMS. The self-developed modules, “ECGHHT” and “MMLCA”, were built using MATLAB^TM^ (license no. 40697750) to progress the featuring and machine learning processes, respectively, for achieving the recognition scope. The computation processes request manual evaluation at the data training stage but achieve automatic processing at the final prediction stage. As training data, the input signals were automatically transformed to the HHT-based features by the ECGHHT module in the slices of data pre-processing and featuring; then, they needed manual evaluation with the MMLCA module in the slices of labeling and machine learning before prediction. The computation skips the slices in manual evaluation and approaches completely automatic mode for prediction.

### 2.1. Data Sampling

**Figure 2:**
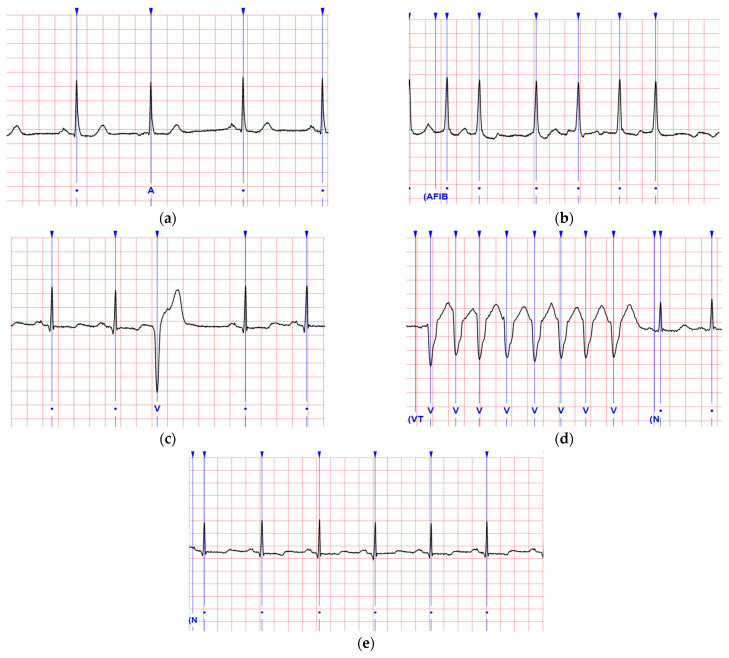
Typical ECG waveforms of the arrhythmia symptoms with annotation by screenshots from MIT-BIH’s web site [18]: (**a**) atrial premature complex (APC); (**b**) atrial fibrillation (AFib); (**c**) ventricular premature complex (VPC); (**d**) ventricular tachycardia (VT); (**e**) normal sinus rhythm (NSR).

The MIT-BIH arrhythmia database’s resource web site [18] presents the ECG data with annotations at the interest time points for various symptoms. We adopted the symptoms, including the normal status (NSR) and the abnormal conditions in the atrium (APC and AFib) and the ventricle (VPC and VT), for sampling in our model. The waveforms of the arrhythmia described above are shown in Figure 2a–e. Their definitions referring to the visible characteristics are addressed below.

#### 2.1.1. Normal Rhythm and Arrhythmia Symptoms

The NSR is the rhythm originating from the sinus node and is observed in a healthy human heart. The NSR presents the heart rate in its normal range, and the P waves are regular on ECGs with conformable rates. The APCs perform premature heartbeats originating in the atria to cause contractions for heart palpitations or unusual heartbeats. The beats of APCs are irregularly timed too fast or slow in an atrial cycle. The AFib is the common arrhythmia that shows the RR intervals following no repetitive pattern and no distinct P waves in an atrial cycle length with a PP interval less than 200 milliseconds. The VPCs are caused by ventricular myocardium situations including patients without structural heart disease. The VT displays a rate greater than 120 beats per minute and at least three vast QRS complexes in a row. The symptom can be classified as sustained versus non-sustained based on whether it lasts more or less than 30 s, respectively.

#### 2.1.2. ECG Waveform Patterns

We noticed the waveform of an AFib would rapidly unite the APC’s waveforms and is similar to a VT that combines with the VPCs. This study, therefore, defined the premature pattern (i.e., APC and VPC) and the fibril-rapid pattern (i.e., AFib and VT) for analysis. In clinical practices, the APC and VPC often appear in ordinary people due to the fact of anxiety and, in such cases, it is not regarded as a severe disease [33]. All points of interest of the specified arrhythmia symptoms annotated on the MIT-BIH website were referred to as the official samples. Their symptom ID labeled the specimens as NSR, APC, AFib, VPC, and VT (i.e., 1, 2, 3, 4, and 5, respectively). The featuring frame, which can contain a complete waveform for different symptoms within a unified period, was applied to screen the samples. For example, the APC and VPC can be mixed with the NSR in a 3 s frame.

According to the MIT-BIH website’s analytical resource for all 48 patients, the 30 min recording was adopted from each patient’s ECG, and several points of interest were annotated for the arrhythmia symptoms (i.e., one point denoted a symptom around the timestamp as shown in Figure 2). One patient’s recording could have some but not all of the five arrhythmia symptoms. We then screened the recording of five patients (#100, #105, #202, #203, and #205), who were observed with many interesting symptoms related to our study. For example, patient #202’s recording was annotated for NSR, APC, AFib, and VPC with 2, 3, 7, and 4 points of interest, respectively; we also observed an additional 507, 14, 278, and 14 points (i.e., we acquired 509, 17, 285, and 18 samples of NSR, APC, AFib, and VPC from patient #202). Finally, we extracted 4190 samples including 176 samples from all patients’ points of interest for NSR (35), APC (11), AFib (23), VPC (24), and VT (83) and 4014 samples self-observed for NSR (2964), APC (97), AFib (649), VPC (272), and VT (32); the total amount of NSR, APC, AFib, VPC, and VT samples were 2999, 108, 672, 296, and 115, respectively. Each sample covered a 3 s frame including a complete waveform of the symptom for a point of interest, and the signals in a frame could be transformed to the features set in a record through HHT processing.

However, the amounts of different symptoms were still insufficient and unbalanced in the observed samples. We then designed three sample data sets below for analysis.

#### 2.1.3. Sample Data Sets

With the resource limitations, we designed crucial, simulative, and test data sets in the modeling. We arbitrarily adopted 300 equivalently labeled data (i.e., 60 records per symptom) for the crucial data set (A) and 3890 remaining records (i.e., 2939, 48, 612, 236, and 55 records for NSR, APC, AFib, VPC, and VT, respectively) for the test data set (B). In addition, we created a simulative data set (C) that had 5000 equivalently labeled data (i.e., 1000 records for each symptom) for comparison. That is, we separated the observed samples into data set (A) and (B) for data training and testing, respectively; then, we created data set (C) with the same amount of the five symptoms, which were produced by normal distribution due to the mean (μ) and standard deviation (σ) of all observed data for the simulation.

In this study, data set (C), independent of the extracted samples but containing an abundant quantity of every arrhythmia feature, supplemented the sufficient and balanced data compared to data set (A) for machine learning. In other words, we applied the feature’s mean and standard deviation, as shown in Table 1, and employed MATLAB’s “normrnd” function that can generate a random data set in normal distribution to create the simulative data set. The data set can randomly fill enough data into the same range as crucial feature’s distribution. We created several simulative data sets for analysis with the randomized process and adopted the appropriate one for demonstration. The data sets (A) and (C) were taken for training data with 5-fold cross-validation in the modeling. This means that 80% and 20% of the data sets were separated for training and validation in each fold to deliver the performance information for modeling evaluation. We tried the cross-validation processes several times to evaluate the proper model, while the training and validating data sets were independent of each other every time. The samples, including the training and testing data points, were applied for the ML models of the MLP, RF, SVM, and NB.

### 2.2. Featuring by Marginal Hilbert Spectrum

We established the module, ECGHHT, to enhance the HHT functions in the featuring process. The HHT drives the EMD process to produce the Hilbert spectrum (HS) for deriving the features in a time–frequency domain. The EMD assumes that the signals are empirically composed of several IMFs that present waveforms with a sum of peaks and troughs equal to one or more of the number of zero-crossing points. In addition, the average of the upper and lower envelopes due to the waveforms should be close to zero. Each EMD cycle, comprising six steps in the “data pre-processing block”, as shown in Figure 1, can obtain an IMF component in the HHT. The steps were repeated until the residue function was monotonic.

The IMF can be expressed by Equation (1) that contains a sine wave function *ω*(*t*) with a decomposable mode function *s_j_*(*t*). The IMF can be converted into the HS by a sparse matrix with the Hilbert transform as shown in Equation (2). The spectrum entails the instantaneous frequency and energy for nonlinear phase change.

(1) $$X(t)=s_{j}\left(t\right)e^{i\int \omega_{j}\left(t\right)dt}$$

(2) $$H\left(\omega ,t\right)=\{\begin{array}{c} s_{j}\left(t\right),\;\;\omega =\omega_{j}\left(t\right)\\ 0,\;\;otherwise \end{array}$$

On the HS’s time–frequency plane, the amplitude of the frequency can be integrated along the time axis to approach the marginal Hilbert spectrum (MHS) as shown in Equation (3). The MHS represents the total energy corresponding to the frequency.

(3) MHS(ω)=∫H(ω,t)dt

**Figure 3:**
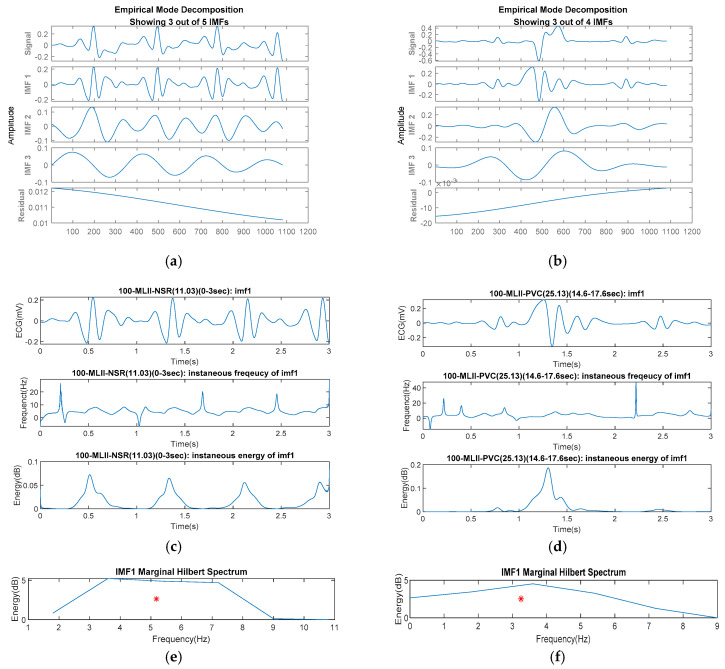
Spectrum analyses in the time–frequency domain due to the HHT process for normal and arrhythmia ECG signals exemplified by NSR and VPC: (**a**) NSR IMFs due to the fact of EMD; (**b**) VPC IMFs due to the fact of EMD; (**c**) NSRs’ instantaneous frequency and energy; (**d**) VPCs’ instantaneous frequency and energy; (**e**) NSR MHS and centroid (red star) for IMF1; (**f**) VPC MHS and centroid (red star) for IMF1.

Therefore, ECG signals can be decomposed by various IMFs that may include the specific symptoms corresponding to arrhythmia. Figure 3a,b compare the IMFs of the NSR and the VPC signals, respectively, decomposed with the HHT process. The instantaneous frequency and energy, as shown in Figure 3c,d, may present the HRV with the waveform of IMF1 (i.e., the first-order IMF). The MHS, as per the examples shown in Figure 3e,f, performs the valid regions and the centroids (i.e., *x*- and *y*-coordinates with respect to frequency and power) of various IMFs in a range of frequency distribution. For this data pre-processing step, the loss-pass filter was employed to filter the noise of the signals for the HHT. Past studies approved a filter at 16 Hz that well-exhibited characteristics of the NSR and arrhythmias [34,35]. Referring to the literature’s approach for the Butterworth filter, the cutoff frequency with a low-pass order between 4 and 8 was one of the suggestive parameters to remove ECG’s noise [36]. We then suggested a 5th-order low-pass digital Butterworth filter with a normalized cutoff frequency of 16 Hz for the data sampled at 360 Hz. The parameter adopted in the modeling must be identical for the processing in recognition. In practice, the proposed parameters should be adjusted once the clinical data applied for training are different from the samples in this study.

The spectral characteristics of IMFs are available for different signal patterns to explore the features in which the instantaneous frequency and energy of the IMFs perform a stable variation for the normal rhythm with respect to the arrhythmias. Meanwhile, the MHS-area centroids (i.e., the mean frequency and the energy per second (or power) in a featuring-frame period) are distributed in the different ranges for various symptoms. We then considered the MHS-area centroids of the first three orders of IMFs as the features for data training in the ML process. In addition, we referred to the mean and standard deviation of the features in the limited data source (i.e., data sets (A) and (B)) to produce sufficient and balanced trainable data (i.e., data set (C)) for evaluating the ML model’s efficacy in different data set groups.

### 2.3. Comprehensive Machine Learning Models

The ML models were bundled into the recognition module, “MMLCA”, for coupling the same HHT-based features in analysis. The MMLCA was expandable to compose comprehensive ML methods, such as the MLP, RF, SVM, and NB, which were well known in biomedical data analysis. We, therefore, adopted them in the modules to recognize the hybrid symptom patterns. For comparison, all modules can be applied to screen the ECG data, including the four arrhythmias. The methodology details can be referred to in the literature [7,8,9,10,11] and their brief highlights are addressed below.

#### 2.3.1. Multiple Layer Perceptron (MLP)

The MLP presents a feedforward neural network to transfer information among the input, hidden, and output layers. The MLP involves enough neuron nodes in a hidden layer for mapping the input-output features of the patterns. The nodes in the hidden and output layers can activate the nonlinear functions to separate the initially heterogeneous data from the linear perceptron. The functions can optimize weight values for bias evaluation, such as backpropagation, regularization, or conjugate gradient descending. Inside the MLP, the learning mechanism can adjust the nodes’ correlation to enhance data training with self-adaptive weighting factors.

#### 2.3.2. Random Forest (RF)

The RF ensembles abundant decision trees that enable non-parametric supervised learning with conditional classification rules for classification. The bagging and boosting algorithms are usually applied for the ensemble analysis according to various sub-samples to enhance the model’s predictive efficacy.

#### 2.3.3. Support Vector Machine (SVM)

The SVM in analysis follows three principles: (a) separating dimensions in the feature space, (b) calculating projection through the inner product of the kernel functions, and (c) optimizing data separation to obtain the support vector. The categorical features could approach good efficacy with analyses due to past research [37]. The method looks for the support vector with an optimized boundary in a feature space due to the kernel function that minimizes the errors between different dimensions. For nonlinear projection, the kernel function is applied to reduce the features’ dimensions through two eigenvectors, such as the linear, polynomial, or Gauss radial basis functions (RBFs), to identify the dimension’s similarity [38,39]. In this study, a RBF was employed to classify the four arrhythmia symptoms and NSR.

#### 2.3.4. Naive Bayes (NB)

The NB drives the independence assumptions conditionally between feature pairs to calculate the probability of features based on Bayes’ theorem. The NB classification can fast estimate the targets with few training data since the parameters can be linearly functioned with the relative variables.

We employed the MATLAB™ modules “patternnet”, “fitcensemble”, “fitcecoc”, and “fitcnb” to wrap up the MLP, RF, SVM, and NB models, respectively, in the MMLCA. The parameters optimized for the ML models are suggestive in Table 2. For enabling the multiclass recognition capability, we compared the coding design modes “one-versus-one” (OVO) and “one-versus-all” (OVA), which were built in the modules to reduce the multiclass problem to a series of binary problems. We then explored the best option with the OVO mode, which takes a binary pair for positive and negative classes but ignores the rest for the approach.

In this study, the recognition model drove the ECGHHT module to extract the HHT-based features and used the MMLCA module to couple the above four ML methods for training the features of the hybrid arrhythmia symptoms.

### 2.4. UHMS Infrastructure

**Figure 4:**
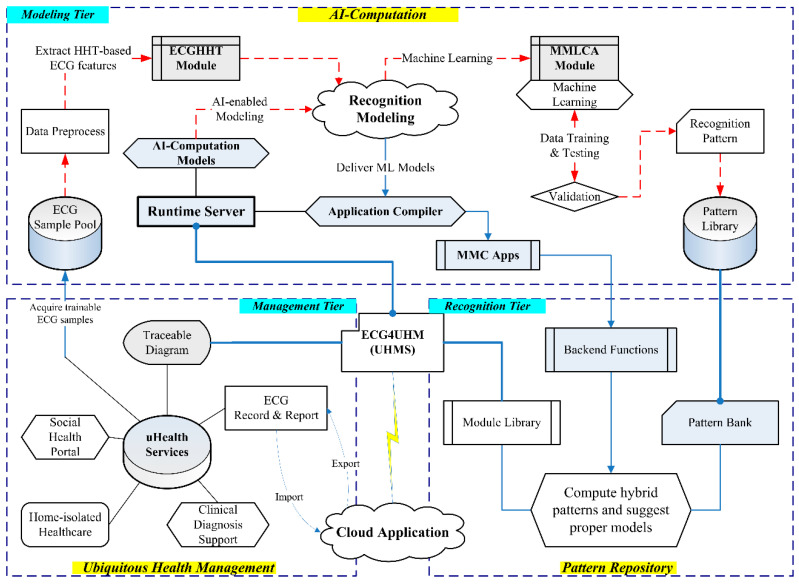
The three-tier UHMS infrastructure for the self-developed ECG4UHM platform with AI computation.

The UHMS infrastructure, as shown in Figure 4, integrates the Java-based web platform “ECG4UHM” with the MATLAB^TM^ runtime server (MRS) to enable AI computation with the proposed ML models for cloud applications in real-time arrhythmia recognition. The modeling and recognition tiers include the dashed and solid arrow lines, respectively, for the manual data training and automatic recognition processes. The platform was constructed by Java development kit 1.8.0 and MATLAB^TM^ 2020b. The infrastructure comprises the modeling, recognition, and management tiers to manage the MRS functions and uHealth services.

#### 2.4.1. Runtime Server Architecture in Modeling Tier

The infrastructure exhibits four AI computation slices, as shown in Figure 1, to generate hybrid patterns based on recognition modeling in the MRS. The runtime server can achieve three procedures in the modeling tier: (i) transform ECG features, (ii) specify recognition patterns, and (iii) identify arrhythmia labels. We engaged the MATLAB™ compiler (named MMC Apps) to compile the MMLCA’s functions, which progress the ML models such as MLP, RF, SVM, and NB for coupling features analysis, such as Java-based classes, and compress them in a Java-class library (named “jar” file). The jar files are stored in ECG4UHM’s pattern library (named “jarlib”). Referring to the MRS architecture, we created two jars named “HHTFeature” and “TracePlot” to incorporate the recognition model with the “javabuilder” that builds Java classes of the modules for AI computation in the MRS. The HHTFeature conducted the ECGHHT and MMLCA modules to accomplish procedures (i) and (ii), and the TracePlot was in charge of the procedure (iii) to export recognition results in the traceable diagrams.

#### 2.4.2. Pattern Repository Design in Recognition Tier

In the ECG4UHM platform, we designed the pattern repository including “data_log”, “patt_bank”, “out_log”, and “fig_log” to save input features, suggestive patterns, recognition labels, and traceable diagrams, respectively. The uploaded ECG data sets are suggested to unique filename with an identified number and timestamp for managing the personal traceable diagram. The ECG4UHM saves the ECG data set in the data_log when identifying the symptoms in the recognition tier. With the Java-class library, the “HHT” class transforms the ECG data into the HHT-based features. The “Pattern-Recognition” class then activates the pattern recognition process by evaluating the suitable models in patt_bank for the diverse arrhythmia symptoms and restores the results in out_log for the suggestion. The “Data-Convert” class finally converts the output labels to Java-compliant data format in fig_log and displays the traceable diagrams on the web interface. The interface supports cloud computing for modeling the pattern and tracking the ECG data behind arrhythmia recognition. Data exchange between the management and recognition tiers assists the users to access their health data ubiquitously.

#### 2.4.3. UHM Portals in Management Tier

The UHMS’s management tier was designed to manage arrhythmia status and daily reports for home-isolated healthcare via uHealth services. The traceable diagram can be retrieved from the ECG4UHM platform and be referred to physicians for clinical diagnoses. More trainable ECG samples can be cyclically uploaded to the modeling tier to improve the recognition patterns for more arrhythmia symptoms. Still, the web page can present information for health education. In advance, the designed UHM functionality can be an important portal of the intelligent social-health system.

## 3. Results

Following the modeling, the ML models due to the crucial data set for the NSR versus both the premature pattern (i.e., APC and VPC) and the fibril-rapid pattern (i.e., AFib and VT) were approved by cross-validation. Then, we applied the models to train data sets (A) and (C) and evaluated their performance by testing data set (B). The range of feature values in the training data set should cover that in the testing data set. The models were finally applied to scan some ECG segments that reported the arrhythmia periods on the official website for evaluating the feasibility.

### 3.1. Pre-Processing Analysis

The HHT process retrieved the features regarding the symptoms of APC, AFib, VPC, VT, and NSR from the sample data sets. From inspecting the features due to the three IMFs of all observed data, the VT’s mean powers were higher than VPC and similar to those observed for AFib versus APC. In contrast, the frequencies of VT and AFib were mainly lower than VPC and APC, respectively. The mean (μ) and standard deviation (σ) of frequencies and powers are shown in Table 1. The NSR usually presented the lowest power and the highest frequency for the three IMFs with respect to other arrhythmia symptoms. The tendency of the orders attained the approach in the past study [40]. In general, the MHS-area centroids of the orders after IMF3 were similar for all rhythms. Regarding the values of μ and σ, the IMF1 had the lower deviation, while the IMF2 and IMF3 had the larger deviation. We then employed a statistical test to verify significance, as shown in Table 1, for various symptoms. The results revealed that the IMF1’s features presented significant difference (i.e., *p* < 0.001) for all patterns excluding AFib–VPC; the IMF2’s frequency was not significant for the patterns APC–VPC, APC–VT, AFib–VPC, AFib–VT, and VPC-VT; while the IMF3’s frequency and power were not significant respectively for the pattern sets {APC–VPC, AFib–VPC, AFib–VT, and VPC–VT} and {AFib–VPC, AFib–VT, and VPC-VT}.

We used the features of data set (A) to train the proposed ML models with five-fold cross-validation, and then the trained models were employed to test data set (B) for evaluation. The same process was applied for data set (C) derived from data set (A) to evaluate the models’ performance due to the simulative data training designs.

### 3.2. Modeling Evaluation

By comparing the ML models’ effectiveness in coupling analysis, we suggested the parameters shown in Table 2 for the proposed models. With the five-symptom set {NSR, APC, AFib, VPC, and VT}, recognition for each symptom versus the rest (i.e., one-versus-rest mode, OVR) was evaluated by the sensitivity, specificity, and area under the curve (AUC) of receiver operating characteristics (ROC) in the modeling. The sensitivity and 1-specificity constructed the ROC curve for the possible cut-point values [41]. The formulations, as shown in Equation (4), comprised the number of true-positive (TP), true-negative (TN), false-positive (FP), and false-negative (FN) data for calculating the accuracy, sensitivity, and specificity due to the ROC to perform the model’s effectiveness. We computed the average accuracy, sensitivity, and specificity of the model’s ROC for evaluation.

Accuracy = (TP + TN)/(TP + FN + FP + TN) Sensitivity = TP/(TP + FN) = TPR (i.e., true positive rate) Specificity = 1 − FP/(FP + TN) = 1 − FPR (i.e., false positive rate)(4)

**Figure 5:**
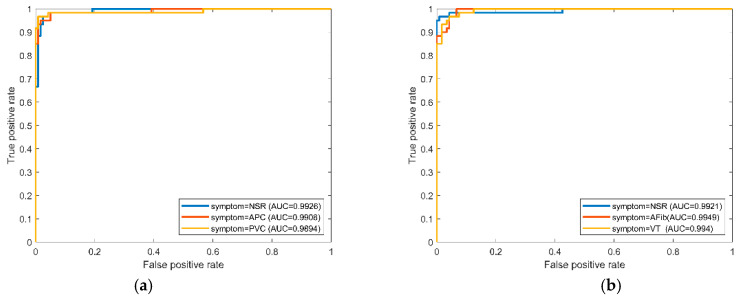
The one-versus-rest ROC curves in the RF model for the NSR versus the premature and fibril-rapid pattern due to the best performance fold in cross-validation by training data set A: (**a**) NSR versus premature pattern (i.e., APC–VPC); (**b**) NSR versus fibril-rapid pattern (AFib–VT).

We compared NSR versus APC and VPC (i.e., premature pattern) in data set (A) trained by the RF model with cross-validation, and the best ROC curves are shown in Figure 5a. The best accuracy and AUC reached 0.99, which can be referred to in the past study [20]. If the NSR mixed with AFib and VT (i.e., the fibril-rapid pattern), the AUC, shown in Figure 5b, was approximately 0.99 on average. The evaluation approved the reliability of the suggested models.

**Figure 6:**
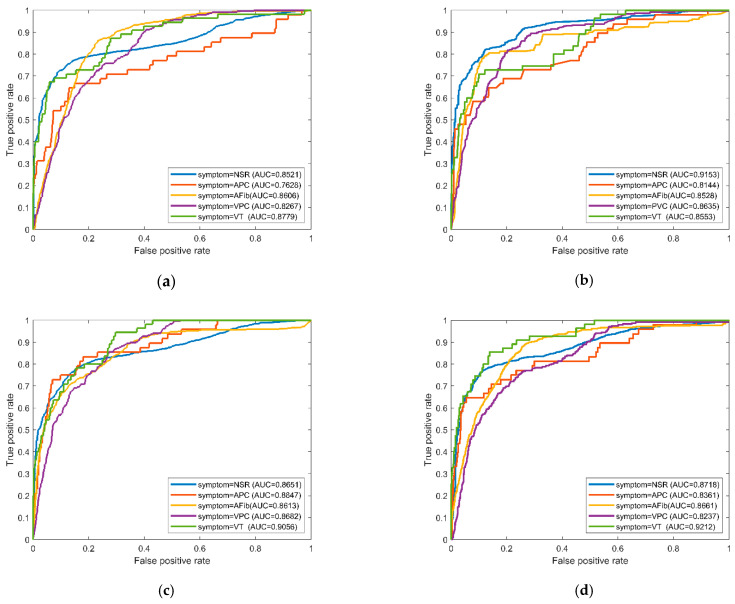
The ROC curves of the arrhythmia symptoms with one-versus-rest plots for the ML models’ coupling analysis by training data set (A) and testing data set (B): (**a**) MLP model; (**b**) RF model; (**c**) SVM model; (**d**) NB model.

Then, we mixed all the patterns for coupling analysis of the proposed ML models. The models were trained by data set A and tested by data set B, and then the recognition results were shown in Table 3. The average accuracy of the four ML models for all hybrid patterns was approximately 86% but reached 90% if excluding VPC versus AFib and VT. The recognition efficacy decreased in comparison with the previous evaluations. Most models distinguished the NSR versus the premature and fibril-rapid patterns with acceptable AUCs (from 0.747 to 0.942). All models validated with the pattern AFib–VT achieved an AUC average of 0.83, but with the AFib–VPC and VPC–VT, the result was less than 0.7. The sensitivities of hybrid patterns, except for VPC–AFib, VPC–VT, and AFib–VT, were over 0.9 for most models. On average, the respective AUCs of the NSR and APC versus AFib, VPC, and VT were 0.94 and 0.93 for the RF and SVM. The MLP, RF, SVM, and NB models’ best sensitivities reached the value of 1 for the APC–VT pattern, while their accuracies and AUCs were {0.964, 1, 0.984, and 0.898} and {0.96, 1, 0.98, 0.89}, respectively. The ensemble analysis of RF obtained good sensitivity, specificity, and AUC for the patterns of NPC and APC versus AFib, VPC, and VT in comparison with the other models. The model performed an excellent sensitivity and specificity of 0.986 and 0.96, respectively, for the NSR–VT, while 0.927 and 0.901 for the NSR–VPC. Figure 6a–d display the OVR ROC curves for all models; the RF and SVM models performed better AUCs over 0.85 on average than other models.

We further evaluated the test results by training the simulative data (i.e., data set (C)) for the proposed models. As shown in Table 4, the average AUCs of all models for both the APC–VPC and AFib–VT patterns versus the NSR could reach approximately 0.84 and 0.92, respectively. The ML models obtained the best AUCs at approximately 0.9 on average for recognizing the patterns of APC–NSR versus AFib–VT. The average accuracy of the MLP, RF, and SVM models for the APC–VT pattern reached approximately 0.983 and 0.977 for both the crucial and simulative data sets, while the respective AUCs were 0.98 and 0.973. However, similar to data set (A), the recognitions among AFib–VPC and VPC–VT were ambiguous. This approach showed the acceptable efficacy of the simulative data compared with the observed data. This implied that the mean and standard deviation of few reliable data could produce sufficient and balanced training data for the ML model.

### 3.3. Implementation

**Figure 7:**
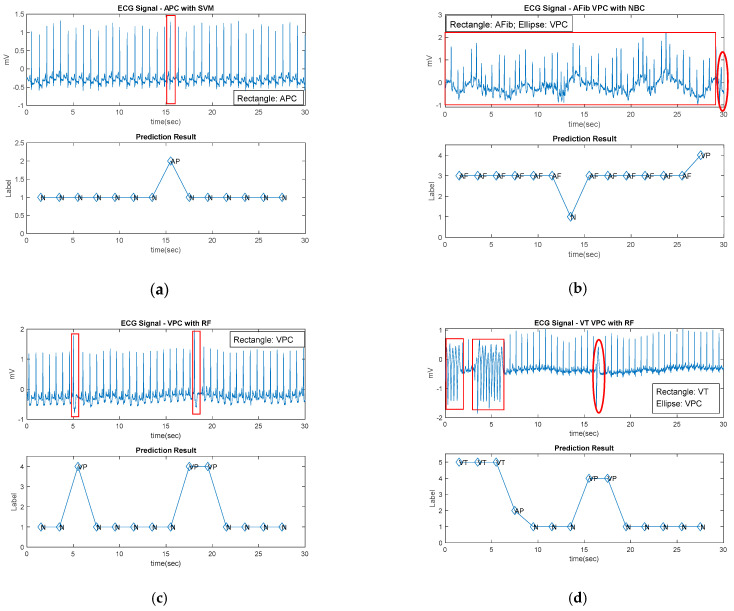
The traceable diagrams of arrhythmia symptoms recognized by the prototype of self-developed programs in comparison with the 30 s ECG segments around the interest timestamp: (**a**) APC by SVM for case #100 at approximately 17:27; (**b**) AFib–VPC by NB for case #203 at approximately 3:00; (**c**) VPC by RF for case #105 at approximately 0:32; (**d**) VT–VPC by RF for case #205 at approximately 5:15.

With the above evaluation, we can suggest the proper models to identify arrhythmia patterns in the UHMS. The proposed modeling was applied to practical cases through the ECGHHT and MMLCA modules. We selected the cases reported to have APC, AFib, VPC, or VT from the official website and screened their records by a 3 s frame with a 1 s overlap during 30 s of an arrhythmia period. The MHS-based features were extracted from each frame by ECGHHT. Then, the labels of APC, AFib, VPC, VT, and NSR could be recognized by MMLCA. Both modules were activated by the HHTFeature built in the ECG4UHM to enable AI computation. The multiclass recognitions of the arrhythmia symptom patterns regarding the original ECG are displayed in Figure 7a–d. Particularly, the NB and RF algorithms recognized the hybrid patterns, including the VPC mixing AFib and VT, as shown in Figure 7b,d, respectively. The traceable diagrams display the ECG signals on the time axis, mapping to the symptom labels highlighted in the associated frames. The modules approved the feasibility of AI computation for the ECG4UHM.

**Figure 8:**
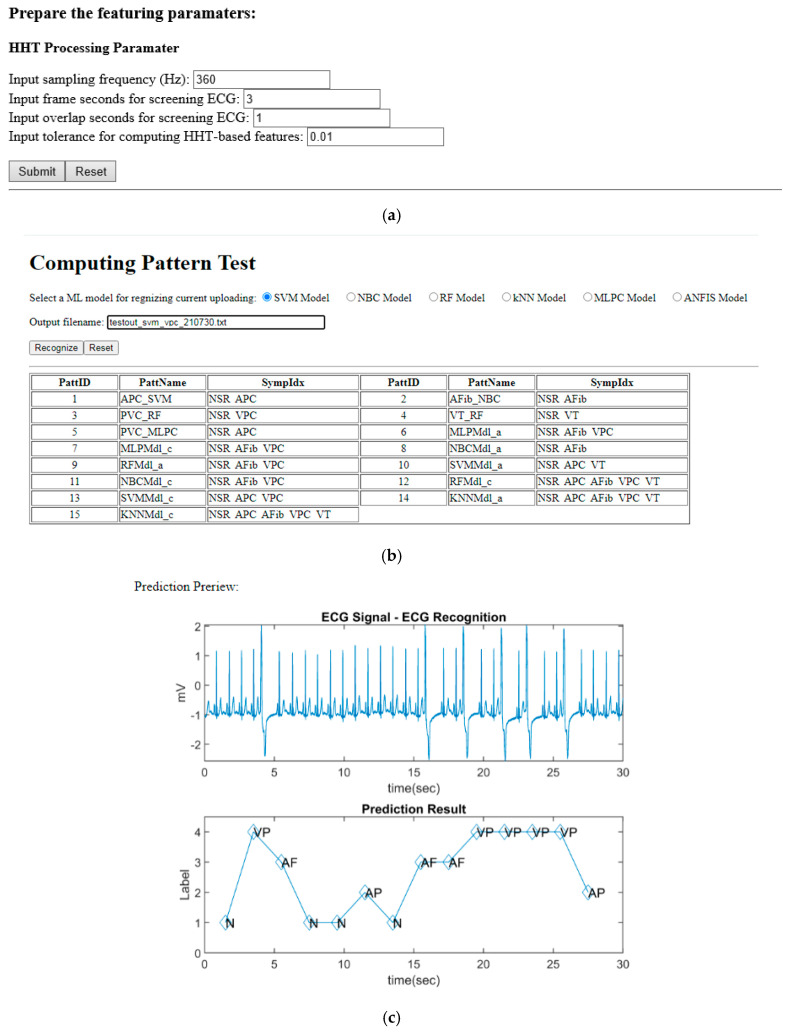

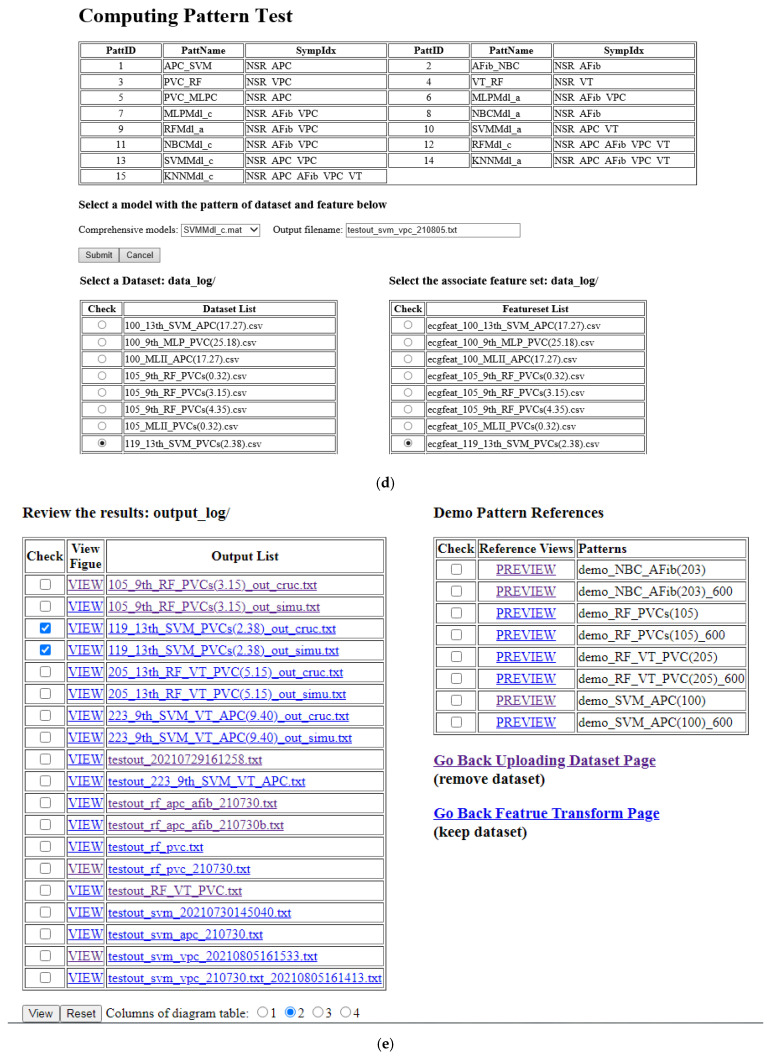

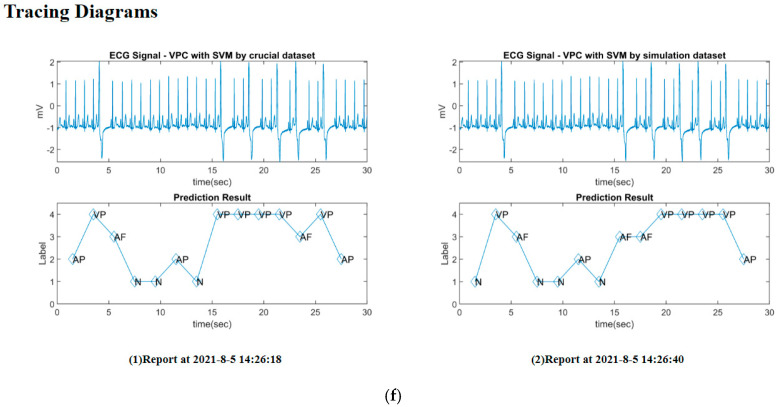
The screen snapshots for recognizing and managing arrhythmia data on the web-based ECG4UHM platform: (**a**) input of the HHT processing parameters for feature extraction; (**b**) selection of the machine learning model for arrhythmia pattern recognition; (**c**) display of the recognition results; (**d**) selection of the input ECG data and correspondent feature set to create the trace diagram; (**e**) management of traceable diagram log for review; (**f**) review page of traceable diagrams.

Furthermore, we practiced the ECG4UHM’s online recognition process. As shown in Figure 8a, the screen snapshot presents the interface for inputting the parameters to transform the uploaded ECG data set into the HHT-based features in the file named by the prefix “feat”. The models available for the various symptoms were suggested for choice as shown in Figure 8b. In addition, the user can compare the comprehensive models to recognize the hybrid arrhythmia patterns according to the ECG data set and the relevant feature set as shown in Figure 8c. The recognized result as shown in Figure 8d was traceable with symptom annotations with the AI computation. All traceable diagrams were saved in the fig_log for advanced management and review as shown in Figure 8e. The implementation approved the feasibility of the UHMS prototype for extensive applications of the uHealth services.

## 4. Discussion

This study suggested four ML models for the UHMS to recognize hybrid arrhythmia ECG patterns. The HHT-based features, including frequency and power due to the MHS of the first three-order IMFs, were coupled with the ML algorithms for analysis. The development of the ECG4UHM detailed the AI computation process, and the prototype revealed the advantages of combining simulative arrhythmia ECGs in the expandable ML modeling. We discuss the findings and limitations below.

### 4.1. Principal Finding

Past studies of HRV applied the spectrum to classify arrhythmias and normal heart rhythm but rarely recognized the symptoms with various pattern waveforms simultaneously [42,43]. Therefore, we discovered the representative features from the HHT-based IMFs after decomposing the ECG signals.

(1) *Candidate features can be extracted from the first three-order IMFs*. In the analysis, we used the low-pass filter to remove noises from the ECG signals before the EMD process. The past study employed this process with the SVM to achieve good performance for recognizing the APC and VPC [20]. As inspecting the decomposed IMFs of the AFib and VT samples, the IMF1 showed the major features of the frequency and power due to the MHS-area centroid in a significant range. At the same time, IMF2 and IMF3 contributed minor characteristics with a dispersed distribution. The MHS-area centroids for the latter-order IMFs were similar for all symptoms. Therefore, the first three-order IMFs, which used to include the hybrid patterns’ recognizable features, are suggested for the candidate features.
(2) *Symptomatic waveforms should be wholly involved in the featuring frame*. The timestamps of the arrhythmia symptoms’ interest points were annotated on the official web page [18], but they mixed with the NSR or other symptoms in a featuring frame. The impure waveforms of the symptoms may affect the features of the observed databases in this study. Therefore, the appropriate sample should involve the complete waveform of the specific symptom in the frame, which allows some NSRs to fill up the frame size and reduce the bias.
(3) *HHT-based data pre-processing can imply innovative features in the ML model.* With EMD in the HHT, the features (i.e., MHS-area centroids for various IMFs) of the multiclass symptoms due to the limited samples were observed to scatter in a separable distribution. The simulative features can be supplemented following the mean and deviation of the observed samples for data training. The evaluation showed similar performances for AFib and VPC corresponding to NSR using the simulative data set compared to the crucial data set. However, the VPC versus AFib and VT did not reach acceptable results, since they lacked enough samples with reliable means and standard deviations for simulation. Various models with ensemble analysis can be pipelined in a suitable pattern for the diverse symptoms to achieve a good recognition in practice. Proper feature pre-processing with validation can avoid unbalanced or insufficient samples and improve training efficacy before constructing the reliable recognition model. This approach revealed the requirement of conventional AI-based analysis in which the significant features can enhance the various machine learning methods to reach good results in classification [44,45,46].
(4) *The coupling ML models can customize the UHMS to recognize hybrid arrhythmia patterns.* The model’s parameters are adjustable for the specific feature set. The current prototype could recognize four arrhythmia symptoms for application and suggest the suitable ML models with respect to the hybrid patterns for the frequency–domain features. The user can determine the most possible symptom based on the models’ suggestion. In advance, we suggested the features in the HS for recognizing more arrhythmia diseases. The HS offers the instantaneous energy and frequency in the time–frequency domain, which also implies the time-dependent characteristics of the HRV symptoms with noticeable phase changes in the wave period. The previous study selected the features in both domains to avoid ambiguous identification for the similar waveform of ventricular arrhythmia [47]. The HS can be reflected as more features when the MHS-area centroids are not apparent than other symptoms [48].

### 4.2. Study Limitation

Some features of arrhythmia symptoms obtained at the current stage were still insufficient for classification. There were only 108, 672, 296, and 115 of the APC, AFib, VPC, and VT, respectively, observed in the sample data sets by limited manpower, which affected the number of equivalent labels of the crucial data set for cross validation as training the data sets. According to the big data concept, the machine learning model would require more training data for a better approach. We increased the supplementary data (i.e., data set (C)) for simulation and comparison. The simulative data were randomly produced by MATLAB’s function and surely located in the same range as the observed sample’s distribution (i.e., data sets (A) and (B)). Three data sets were ensured in the same distribution but independent for training, validation, and testing in machine learning.

In practice, the arrhythmia patients could report their experienced symptoms (e.g., chest pain, racing heartbeats, and shortness of breath) to the clinicians. The AI system developed at the current stage could not identify these self-reported conditions but only helped classify arrhythmia due to the ECG patterns. From the aspect of ML modeling, the sample data set could involve the measured ECG features and the reported symptoms in the clinical records for the advanced data training process (e.g., let AFib’s features contain the label of chest pain). The improved model could be more feasible in clinical application.

The modeling’s effectiveness can be improved if more useful features and eligible samples of the various symptoms are classified. Currently, mobile ECG apps are restricted in some countries. Therefore, the prototype design in this study contributed to a portal of sensing application to connect the ECG sensors with biomedical signal processing for the uHealth services.

### 4.3. Comparison with Prior Work

In practice, the multiclass discrimination usually contains unbalanced and high-dimensional data clusters. The previous studies suggested the concept of complexity reduction in the SVM and NN-based modeling to improve convergence efficiency [49,50]. The simulation implied that the multiple labels of the APC, AFib, VPC, and VT versus NSR could be reduced to the pairs of binary classes to achieve the model with reliable sensitivity and specificity. Some prior works merged the similar-property symptoms in a class, e.g., {supraventricular tachycardia, atrial tachycardia, sinus tachycardia} and {atrial flutter, atrial fibrillation} to increase the model’s recognition capability [21]. In addition, the standard deviations of the HHT-based feature distribution, as shown in Table 1, revealed the overlaps between the features of the various symptoms, which could cause ambiguous separation between the multiple classes and lead to difficulty in discrimination. We then verified the statistical significance in various features and compared their *p*-values with the overlapped level. The statistical test performed *p*-value less than 0.001 for each feature with respect to the five symptoms in the OVR mode. We further applied the test of multiple categories for each feature corresponding to the symptom patterns in the OVO mode and showed all *p*-values in Table 1. The patterns with *p*-values greater than 0.05 (i.e., non-significant difference) implied poor recognitions evaluated by ROC data as shown in Table 3. The test could approve the proper model including the feature patterns with significant difference. Therefore, we experienced that the physicians’ knowledge was necessary in clinics to exclude the outliers and uncertain data to ensure the cleanup sample in the modeling. For diagnosing assistance, the multiclass recognition modeling at the current stage can distinguish hybrid patterns of the arrhythmia symptoms against the normal heart rhythms.

Many health service developers keep in customizing a user-friendly interface for commercial application. The customization process due to the wearable device’s requirement is a major challenge for popular home-use solutions. The ML model’s development is still undergoing to improve the UHM services with appropriate interfaces for advanced implementation. In the next phase, we will consider the deep learning methods with pre-processing of diverse arrhythmia ECG signals in the time–frequency domain through HHT to improve the developed programs’ predictability.

## 5. Conclusions

This study proposed a prototype of the self-developed UHMS with the ML modeling for AI computation to recognize arrhythmia ECG in uHealth services. The studied arrhythmia symptoms mixed the premature pattern (i.e., APC and VPC) and the fibril-rapid (i.e., AFib and VT) at the current stage. The MIT-BIH arrhythmia open database was applied for the modeling. We observed the limited data points from the MIT-BIH website’s records for the training and testing processes. The modeling retrieved the features from the IMF through the EMD process based on the HHT algorithm. The HHT-based features included the frequency and power following the MHS-area centroids of the first three-order IMFs. Four ML models, including MLP, RF, SVM, and NB, were trained for coupling features analysis. The ML models’ efficacy achieved the good AUCs of ROC by separately recognizing the premature and fibril-rapid patterns of multiple symptoms. All models could reach the perfect sensitivity for the pattern APC–VT but recognizing the AFib–VPC and VPC–VT requires improvement. The suggestive models were engaged to the UHMS platform “ECG4UHM” as the backend for tracking the arrhythmia symptoms in the real-time UHM services. The development can be integrated with the intelligent social-health system for the home-isolated cares in the future.

## Figures and Tables

**Table 1:** HHT-based feature distribution of the observed samples for the symptoms.

Symptoms	NSR	APC	AFib	VPC	VT
**Features**	IMF1 (μ, σ)				
Frequency	4.772, 0.787	4.241, 0.798	3.571, 0.558	3.283, 0.973	3.120, 1.123
Power	1.167, 0.864	0.791, 0.567	2.683, 1.214	3.196, 2.170	6.504, 6.250
**Features**	IMF2 (μ, σ)				
Frequency	1.478, 0.527	0.932, 0.504	1.049, 0.455	0.993, 0.503	1.013, 0.489
Power	1.344, 1.605	1.223, 1.294	2.824, 2.053	4.548, 3.909	6.923, 7.274
**Features**	IMF3 (μ, σ)				
Frequency	0.288, 0.261	0.378, 0.257	0.327, 0.263	0.340, 0.264	0.315, 0.265
Power	0.645, 1.997	1.461, 2.762	1.183, 2.024	1.449, 2.011	1.184, 1.070
**IMF1’s Frequency**		**IMF2’s Frequency**		**IMF3’s Frequency**	
**Pattern**	***p*-Value**	**Patterns**	***p*** **-Value**	**Pattern**	***p*** **-Value**
NSR–APC	<0.001	NSR–APC	<0.001	NSR–APC	0.0003
NSR–AFib	<0.001	NSR–AFib	<0.001	NSR–AFib	0.0009
NSR–VPC	<0.001	NSR–VPC	<0.001	NSR–VPC	0.006
NSR–VT	<0.001	NSR–VT	<0.001	NSR–VT	0.3482
APC–AFib	<0.001	APC–AFib	0.0286	APC–AFib	0.037
APC–VPC	<0.001	APC–VPC	0.3926	APC–VPC	0.1081
APC–VT	<0.001	APC–VT	0.2015	APC–VT	0.0407
AFib–VPC	<0.001	AFib–VPC	0.0871	AFib–VPC	0.6571
AFib–VT	<0.001	AFib–VT	0.5691	AFib–VT	0.6032
VPC-VT	0.0302	VPC-VT	0.6266	VPC-VT	0.4894
**IMF1’s Power**		**IMF2’s Power**		**IMF3’s Power**	
**Pattern**	***p*** **-Value**	**Pattern**	***p*** **-Value**	**Pattern**	***p*** **-Value**
NSR–APC	<0.001	NSR–APC	0.4453	NSR–APC	<0.001
NSR–AFib	<0.001	NSR–AFib	<0.001	NSR–AFib	<0.001
NSR–VPC	<0.001	NSR–VPC	<0.001	NSR–VPC	<0.001
NSR–VT	<0.001	NSR–VT	<0.001	NSR–VT	<0.001
APC–AFib	<0.001	APC–AFib	<0.001	APC–AFib	0.0024
APC–VPC	<0.001	APC–VPC	<0.001	APC–VPC	0.0024
APC–VT	<0.001	APC–VT	<0.001	APC–VT	0.0052
AFib–VPC	0.3851	AFib–VPC	<0.001	AFib–VPC	0.4177
AFib–VT	<0.001	AFib–VT	<0.001	AFib–VT	0.3471
VPC-VT	<0.001	VPC-VT	0.0028	VPC-VT	0.7985

**Table 2:** Essential parameters of the ML models in data training.

ML Model	Parameter	Value
MLP	hidden layer size	10
	backpropagation training function	scaled conjugate gradient
	performance validation function	cross entropy
RF	ensemble aggregation method	adaptive boosting
	learning cycles	100
	nodes in trees	10
SVM	kernel function	linear
	coding design	OVO
	estimation output	posterior probability
	kernel scale parameter	1
NB	distribution for the nodes	Gaussian distribution
	smoothing density support	real values

**Table 3:** Evaluation of the ML models for the patterns of hybrid symptoms due to the training the data set (A) and testing the data set (B), which the pattern labeled by two symptoms (e.g., NSR–APC means normal sinus rhythm versus atrial premature atrial complex) presents their ROC’s results due to the four models (i.e., multiple layer perceptron, random forest, support vector machine, and naive Bayes models) for comparison.

Pattern	ROC	MLP	RF	SVM	NB
NSR–APC	Sensitivity	0.842	0.782	0.774	0.815
	Specificity	0.857	0.837	0.907	0.889
	Accuracy	0.842	0.783	0.777	0.816
	AUC	0.85	0.81	0.84	0.85
NSR–AFib	Sensitivity	0.913	0.916	0.941	0.812
	Specificity	0.918	0.931	0.861	0.91
	Accuracy	0.913	0.919	0.929	0.83
	AUC	0.92	0.92	0.9	0.86
NSR–VPC	Sensitivity	0.9	0.927	0.953	0.958
	Specificity	0.673	0.901	0.683	0.657
	Accuracy	0.89	0.925	0.936	0.943
	AUC	0.79	0.91	0.82	0.81
NSR–VT	Sensitivity	0.959	0.986	0.989	0.997
	Specificity	0.857	0.96	0.8	0.955
	Accuracy	0.957	0.985	0.986	0.996
	AUC	0.91	0.97	0.89	0.98
APC–AFib	Sensitivity	0.882	0.878	0.907	0.78
	Specificity	0.844	0.929	0.89	0.957
	Accuracy	0.847	0.925	0.982	0.943
	AUC	0.86	0.9	0.9	0.87
APC–AFib	Sensitivity	0.769	1	0.975	0.914
	Specificity	0.826	0.862	0.826	0.784
	Accuracy	0.809	0.895	0.865	0.821
	AUC	0.8	0.93	0.9	0.85
APC–VT	Sensitivity	1	1	1	1
	Specificity	0.923	1	0.96	0.778
	Accuracy	0.964	1	0.984	0.898
	AUC	0.96	1	0.98	0.89
AFib–VPC	Sensitivity	0.724	0.793	0.742	0.863
	Specificity	0.521	0.613	0.714	0.445
	Accuracy	0.676	0.75	0.736	0.77
	AUC	0.62	0.7	0.73	0.65
AFib–VT	Sensitivity	0.899	0.915	0.888	0.992
	Specificity	0.667	0.828	0.889	0.656
	Accuracy	0.879	0.909	0.888	0.97
	AUC	0.78	0.87	0.89	0.82
VPC–VT	Sensitivity	0.673	0.685	0.709	0.726
	Specificity	0.649	0.490	0.533	0.568
	Accuracy	0.667	0.636	0.665	0.682
	AUC	0.66	0.59	0.62	0.65

**Table 4:** Evaluation of the ML models for the patterns of hybrid symptoms due to the training the data set (C) and testing the data set (B), which the pattern labeled by two symptoms (e.g., NSR–APC means normal sinus rhythm versus atrial premature atrial complex) presents their ROC’s results due to the four models (i.e., multiple layer perceptron, random forest, support vector machine, and naïve Bayes models) for comparison.

Pattern	ROC	MLP	RF	SVM	NB
NSR–APC	Sensitivity	0.917	0.819	0.903	0.859
	Specificity	0.789	0.833	0.821	0.825
	Accuracy	0.917	0.819	0.902	0.859
	AUC	0.85	0.83	0.86	0.84
NSR–AFib	Sensitivity	0.887	0.907	0.905	0.906
	Specificity	0.815	0.82	0.787	0.808
	Accuracy	0.876	0.895	0.888	0.889
	AUC	0.85	0.86	0.85	0.86
NSR–VPC	Sensitivity	0.961	0.97	0.932	0.969
	Specificity	0.629	0.65	0.652	0.595
	Accuracy	0.945	0.953	0.918	0.951
	AUC	0.8	0.81	0.79	0.78
NSR–VT	Sensitivity	0.994	0.995	0.994	0.996
	Specificity	0.793	0.84	0.806	0.786
	Accuracy	0.992	0.994	0.992	0.993
	AUC	0.89	0.92	0.9	0.89
APC–AFib	Sensitivity	0.811	0.921	0.842	0.868
	Specificity	0.952	0.722	0.929	0.913
	Accuracy	0.94	0.738	0.92	0.909
	AUC	0.88	0.82	0.89	0.89
APC–AFib	Sensitivity	0.909	0.921	0.914	0.917
	Specificity	0.886	0.757	0.898	0.821
	Accuracy	0.893	0.801	0.9	0.85
	AUC	0.9	0.84	0.91	0.87
APC–VT	Sensitivity	1	1	1	1
	Specificity	1	0.875	0.962	0.957
	Accuracy	1	0.949	0.983	0.982
	AUC	1	0.94	0.98	0.98
AFib–VPC	Sensitivity	0.805	0.776	0.692	0.849
	Specificity	0.527	0.553	0.662	0.473
	Accuracy	0.741	0.719	0.685	0.759
	AUC	0.67	0.66	0.68	0.66
AFib–VT	Sensitivity	0.963	0.976	0.921	0.978
	Specificity	0.719	0.75	0.735	0.667
	Accuracy	0.945	0.958	0.905	0.955
	AUC	0.84	0.86	0.83	0.82
VPC–VT	Sensitivity	0.673	0.685	0.709	0.726
	Specificity	0.649	0.490	0.533	0.568
	Accuracy	0.667	0.636	0.665	0.682
	AUC	0.66	0.59	0.62	0.65

## Data Availability

Not applicable.

## Abbreviations

AFib	atrial fibrillation
APC	atrial premature atrial complex
AUC	area under the curve of receiver operating characteristics
HHT	Hilbert–Huang transform
MHS	marginal Hilbert spectrum
MLP	multiple layer perceptron
NB	naive Bayes
NSR	normal sinus rhythm
RF	random forest
ROC	receiver operating characteristics
SVM	support vector machine
VPC	ventricular premature complex
VT	ventricular tachycardia
